# The Copenhagen Neck Functional Disability Scale: an analysis of singers with dysphonia and without vocal complaints

**DOI:** 10.1590/2317-1782/20212021095en

**Published:** 2022-07-25

**Authors:** Thalita Ferreira Rodrigues Lopes, Bárbara Pereira Lopes Lobo, Ana Cristina Côrtes Gama

**Affiliations:** 1 Departamento de Fonoaudiologia, Universidade Federal de Minas Gerais – UFMG - Belo Horizonte (MG), Brasil.

**Keywords:** Voice, Dysphonia, Voice Disorders, Singing, International Classification of Functioning, Disability and Health, Quality of Life, Neck Pain, Muscle Strength

## Abstract

**Purpose:**

To analyze and compare the degree of cervical disability in singers with dysphonia and in singers without self-reported vocal complaints.

**Methods:**

A cross-sectional observational analysis. Sixty-two singers participated in the study: Thirty-two singers without vocal complaints and 30 singers with a speech-language pathology and otorhinolaryngological diagnosis of dysphonia. For singers without vocal complaints, two questionnaires were applied via Google Forms: A three-question questionnaire regarding vocal complaints and how each singer perceived their speaking and singing voice, and the Copenhagen Neck Functional Disability Scale (CNFDS). Data on singers with dysphonia were extracted from a database previously collected by a team of researchers at the speech-language pathology department of the Federal University of Minas Gerais. The difference in data collection methodology between the two groups was due to restrictions presented by the coronavirus pandemic. The Mann-Whitney test was used to compare the two groups, at a significance level of 5%.

**Results:**

There was a significant difference between the groups (p=0.0001), demonstrating that singers with dysphonia suffered more from cervical pain and discomfort than singers without vocal complaints.

**Conclusion:**

Singers with dysphonia have more cervical pain and discomfort than singers without vocal complaints, thus presenting with greater cervical disability.

## INTRODUCTION

The *Copenhagen Neck Functional Disability Scale (CNFDS)* is a Danish questionnaire that assesses the extent to which neck pain impairs an individual's life and daily activities^(1)^. The CNFDS is easy to apply, does not require the presence and intervention of a professional, and is a practical tool with excellent reliability^(2)^.

The tool was translated and culturally adapted to Brazilian Portuguese, and called *Escala Funcional de Incapacidade do Pescoço de Copenhagen – EFIPC* ^(1)^.

Vocal changes impact individuals' quality of life. When the voice is used professionally, the impact is even more significant, independent of the degree of dysphonia^(3)^. Therefore, voice problems may impact, even minimally, people's quality of life and professional performance^(4)^.

Some cervical region disorders may cause pain and affect the functional performance of various muscle groups and systems, including the anterior neck muscles and the larynx. This may consequently result in vocal tract and voice production changes^(5,6)^. Concomitantly, studies show that certain types of dysphonia may also be related to muscle changes that may cause discomfort and neck pain^(6)^.

Neck muscles are directly related to extrinsic and intrinsic muscles of the larynx, causing vocal changes and cervical function changes in situations of excessive and repetitive phonatory effort^(7)^.

Cervical muscle pain and tension directly affect the extrinsic muscles of the larynx and consequently alter the intrinsic muscles of the larynx. As such, the vocal folds' structure and function are altered, causing individuals to suffer some form of dysphonia^(8,9)^.

In addition, the literature^(10)^ shows evidence that tension in the shoulder girdle region and neck pain are common complaints in individuals with dysphonia, demonstrating that these musculoskeletal alterations are more present in individuals with dysphonia than in individuals without vocal complaints.

Muscle Tension Dysphonia (MTD)^(7)^ is defined as a change in voice quality due to excessive phonatory effort. This phonatory effort is characterized as a compensatory muscular hyperfunction that alters laryngeal, articulatory, resonant, respiratory, and cervical functionality, and performance, leading to MTD ^(7)^.

Many factors can trigger MTD, especially abusive vocal behavior, turning it into a compensatory laryngeal disorder^(11)^.

The literature^(12)^ suggests being attentive in looking for factors associated with vocal and laryngeal symptoms in individuals with dysphonia. However, there are only a few studies showing a correlation between dysphonia and neck pain, despite it being a frequent clinical symptom found in individuals with voice disorders^(12,13)^.

Research considers voice professionals with dysphonia as a group with more neck pain and discomfort complaints than voice professionals without vocal complaints^(13)^. Voice professionals are generally more susceptible to developing negative vocal and cervical signs and symptoms. This is due to greater vocal demand, greater respiratory demand, and specific body posture, according to the type of professional activity^(14)^.

Furthermore, studies suggest that individuals with dysphonia present more constant and intense cervical region discomfort and neck pain compared to individuals without vocal complaints, which play a greater impact on their overall quality of life^(10)^.

The purpose of this study was to analyze whether singers with dysphonia presented with a higher occurrence of cervical discomfort and pain complaints compared to singers with no self-reported vocal complaints.

## METHODS

This was a cross-sectional observational analysis study, approved by the Research Ethics Committee of the Federal University of Minas Gerais (UFMG), under protocol number 59014916.6.0000.5149.

Subjects were sixty-two (62) singers divided into two groups:

1. Group 1 (G1): Singers with dysphonia, characterized by the presence of vocal alterations as defined by a speech-language pathology evaluation, and by the presence of laryngeal alterations as defined by an ENT evaluation (n=30);
2. Group 2 (G2): Singers with no self-reported vocal complaints (n=32).

Inclusion criteria were to be a pop singer, amateur or professional, between 18 and 55 years of age. For G1, the inclusion criteria further included alterations in voice quality, vocal complaints, and laryngeal alterations. For G2, the inclusion criteria further included no complaints of voice alterations and vocal symptoms.

Exclusion criteria included being in the menstrual or premenstrual period, being pregnant at the time of data collection, and/or presence of upper airway infections.

Auditory-perceptual voice analysis was performed by two speech-language pathologists with more than 10 years of voice experience to assess the voice quality of G1. The general degree of dysphonia was the auditory-perceptual parameter analyzed on a four-point scale, with zero (0) for neutral voice quality and three (3) for intense alteration.

For the ENT evaluation, G1 participants underwent a high-speed videoendoscopy (HSV) exam performed with the following equipment: The Color High-Speed Video System (CHSV), model 9710 by KayPentax® (Kay Elemetrics Corporation, Lincoln Park, NJ, USA). Exams were performed and analyzed by two ENT physicians. Laryngeal alterations were defined on the basis of glottic closure alterations and/or vocal fold lesions.

To analyze the presence or absence of self-reported voice complaints by both groups of singers (G1 and G2), three voice self-assessment questions were provided:

1. Do you have any difficulties or discomfort in your singing voice?
2. Do you have any difficulties or discomfort in your speaking voice?
3. Do you think that your voice has changed?

Vocal complaints were considered present as defined by subjects answering affirmatively to the three questions. Vocal complaints were considered absent as defined by subjects answering negatively to the three questions.

G1 was composed of 30 singers with dysphonia, between 21 and 54 years of age (mean=30.33, SD=7.17). Eighteen (60.0%) were amateur singers and twelve (40.0%) were professional singers. Twenty-six presented with a mild degree of voice quality alteration, while four presented with a moderate degree of voice quality alteration. The laryngeal evaluation revealed nineteen singers (63.33%) with the following diagnoses: Mid-posterior triangular glottic gap (N=9); vocal fold nodules (N=7); vocal fold cyst (N=2) and anterior glottic gap (N=1).

G2 was composed of 32 singers with no self-reported vocal complaints, between 19 and 55 years of age (mean= 29.1, SD= 9.89). Seventeen (53.1%) were amateur singers and fifteen (46.9%) were professional singers.

For the purpose of this study, professional singers were considered individuals who were paid for their performance activities^(15)^. The groups were matched by age, with no statistical difference between them (p=0,13).

### The Copenhagen Neck Functional Disability Scale (CNFDS)

The CNFDS questionnaire is a self-assessment protocol, adapted to Brazilian Portuguese^(16)^. It is composed of 15 neck pain-related questions, five questions with a positive direction, and 10 questions with a negative direction. The CNFDS was used in this study to assess and measure the extent to which neck pain was harmful to the singers' quality of life, voice function, and daily performance.

Questions 1 to 5 of the questionnaire were of positive direction, which meant that if singers answer “yes”, they presented with an adequate cervical condition. For these questions, the answers were scored as follows: The option “yes” corresponded to 0 points; “sometimes” corresponded to 1 point, “no” corresponded to 2 points, and “does not apply” corresponded to no value (-). Questions 6 to 15 were questions of negative direction, which meant that if singers answer “yes”, they presented with an inadequate cervical condition. For these questions, the answers were scored as follows: The option “yes” corresponded to 2 points, “sometimes” corresponded to 1 point, “no” corresponded to 0 points, and “does not apply” corresponded to no value (-). The maximum CNFDS score was 30 points. The higher the score, the greater the cervical dysfunction^(16)^.

Singers with dysphonia (G1) signed a Free and Informed Consent Form and answered the vocal complaint questions and the CNFDS in person. Singers with no self-reported vocal complaints (G2) signed a Free and Informed Consent Form and answered the two questionnaires digitally, using the online tool *Google Forms*. The difference in the application of the questionnaires between the two groups was due to the beginning of the coronavirus pandemic (SARS-COV-2).

### Statistical analysis

Statistical analysis of the data was performed using the MINITAB statistics program, version 17. A descriptive analysis of the data was performed with measures of central tendency and dispersion. The Anderson-Darling test was used to verify the normality of the sample. The non-parametric Mann-Whitney test was used to compare groups. A 95% confidence level was considered.

## RESULTS

Table 1 presented the CNFDS data of both analyzed groups: Singers with dysphonia and singers with no self-reported vocal complaints. Results indicated that singers with dysphonia obtained a higher final score on the questionnaire compared to singers with no self-reported vocal complaints. Findings suggested that singers with dysphonia presented with more complaints, and functional and daily limitations due to neck pain.

**Table 1:** Comparison of the total score of the CNFDS questionnaire between both groups

	Minimum	Maximum	Average	Median	SD	p-value
G1	0.0	24.0	7.4	6.0	5.8	
G2	0.0	14.0	2.7	1.0	4.05	0.0001

**Caption:** G1 – Singers with dysphonia; G2 – Singers with no self-reported vocal complaints; SD – Standard Deviation

## DISCUSSION

Dysphonia may be associated with laryngeal discomfort that may alter phonation, in addition to causing emotional and social discomfort, interfering with individuals' daily activities ^(15-17)^.

MTD is considered multifactorial in nature^(11)^, and it may be associated with muscle hyperfunction to compensate for glottal insufficiency, requiring greater effort and vocal compensation during phonation. Studies^(13,14)^ revealed that professional voice users may present with many negative vocal signs and symptoms due to intense voice demand, which may lead to vocal fatigue and, consequently, to greater cervical tension and muscle pain due to negative compensatory behaviors.

Women tend to present with a higher chance of developing vocal alterations and cervical problems due to smaller laryngeal anatomy associated with a higher-pitched voice^(12)^.

The results of this study showed that singers with dysphonia presented with more complaints and daily functional limitations due to neck pain when compared to singers with no self-reported vocal complaints. The literature supported these results as studies showed that women with dysphonia reported more laryngeal, cervical, and back pain than women with no vocal complaints^(6)^. Furthermore, studies^(6,12)^ suggested that women with dysphonia tend to have cervical muscle shortening due to muscle hyperfunction and dysphonia, caused by voice misuse and abuse^(6,12)^.

Voice production changes may lead to dysfunction of the anterior neck muscles, such as the supra and infrahyoid, scalene, and sternocleidomastoid muscles^(5)^. Individuals with dysphonia may have neck pain both at rest and during phonation, in addition to overall greater muscular hyperactivity involved in voice production^(18)^, resulting in postural changes and reduced shoulder girdle movements^(19,20)^. Cervical postural changes can alter the position of the head and neck, leading to functional regional changes. These changes may lead to changes in the vocal tract and, consequently, changes in vocal quality^(21,22)^.

Voice is essential for professional voice users^(23)^, especially in the case of artistic voice professionals, as they present with greater vocal demand and more refined adjustments in their production^(3)^. Adequate cervical disability assessment in singers with dysphonia is important in order to appropriately direct the vocal therapeutic process.

Limitations to this study included the fact that the inclusion criterion for G2 was characterized only by the presence/absence of vocal complaints in the speaking and/or singing voice. This limitation was due to the coronavirus pandemic restrictions, which limited in-person data collection. Studies suggested that singers have a refined vocal self-perception and a greater awareness and concern for their voices^(24)^. Therefore, even a mild voice disorder may become significant enough to affect the quality of speech and overall life and occupation, hence a greater presence of self-reported vocal complaints^(24)^. In addition, singers are able to report in detail the perceived vocal and body changes, because they use their voices daily as a professional instrument^(3,13,14)^.

Another limitation was that participants were not asked if they played a musical instrument, which may impact their body posture and trigger changes and cervical complaints. According to the literature, 84% of musicians report having some type of musculoskeletal complaint^(25)^. It is also noteworthy that this study evaluated and showed an association between dysphonia and cervical complaints in singers with and without dysphonia. However, further studies are needed to verify this causal relationship.

Self-assessment protocols are typically not perfect classifiers and should not be used in isolation. However, the CNFDS proved to be a useful instrument with good applicability in the voice clinic and may contribute to the assessment of cervical functionality in singers with dysphonia.

## CONCLUSION

This study demonstrated that singers with dysphonia experienced more cervical pain and discomfort than singers with no self-reported vocal complaints, thus presenting with greater cervical disability.
