# Supplementary material for: The Copenhagen Neck Functional Disability Scale: an analysis of singers with dysphonia and without vocal complaints
Source: Codas. 2022 Jul 25;34(6):e20210095. doi: 10.1590/2317-1782/20212021095en (PMC9886291; doi:10.1590/2317-1782/20212021095en)
Supplement: Translation

# Translation: Escala Funcional de Incapacidade do Pescoço de Copenhagen: análise em cantoras disfônicas e em cantoras sem queixas vocais

**DOI:** 10.1590/2317-1782/20212021095pt

**Authors:** Thalita Ferreira Rodrigues Lopes, Bárbara Pereira Lopes Lobo, Ana Cristina Côrtes Gama

**Affiliations:** 1 Departamento de Fonoaudiologia, Universidade Federal de Minas Gerais – UFMG - Belo Horizonte (MG), Brasil.

**Keywords:** Voz, Disfonia, Distúrbios da Voz, Canto, Classificação Internacional de Funcionalidade, Incapacidade e Saúde, Qualidade de Vida, Dor Cervical, Força Muscular

## Abstract

**Objetivo:**

analisar e comparar o grau de incapacidade cervical em cantoras disfônicas e em cantoras sem queixas vocais autorreferidas.

**Método:**

estudo observacional analítico transversal. Participaram do estudo 32 cantoras sem queixas vocais e 30 cantoras com diagnóstico fonoaudiológico e otorrinolaringológico de disfonia. Para as cantoras sem queixas vocais foram utilizados dois questionários aplicados virtualmente via *GoogleForms*, sendo um questionário composto por três perguntas referentes às queixas vocais e como cada cantora percebia sua voz de falada e de forma cantada e a Escala Funcional de Incapacidade do Pescoço de Copenhagen (EFIPC). Os dados referentes às cantoras disfônicas foram extraídos de um banco de dados previamente coletado por uma equipe de pesquisadores no Observatório de Saúde Funcional em Fonoaudiologia da Universidade Federal de Minas Gerais e a diferença na metodologia de coleta de dados entre os dois grupos deu-se devido às restrições apresentadas pela pandemia causada pelo coronavírus. Para a comparação entre os dois grupos, foi utilizado o teste Mann-Whitney e foi considerado um nível de significância de 5%.

**Resultados:**

Houve diferença significativa entre os grupos (p=0,0001), demonstrando que as cantoras disfônicas sofrem mais com dores e desconfortos cervicais do que as cantoras sem queixas vocais.

**Conclusão:**

cantoras disfônicas apresentam mais dor e desconforto cervical do que as cantoras sem queixas vocais, caracterizando uma maior incapacidade cervical.

## INTRODUÇÃO

O *Copenhagen Neck Functional Disability Scale (CNFDS)* é um questionário dinamarquês que avalia em que proporção a cervicalgia prejudica a vida e as atividades diárias do indivíduo^(1)^. O *CNFDS* é de fácil aplicação, não exige a presença e a intervenção de um aplicador e, é uma ferramenta prática e de excelente confiabilidade^(2)^.

A necessidade de utilizar essa ferramenta no Brasil fez com que ela fosse traduzida e adaptada culturalmente para o português brasileiro, chegando à uma versão final do questionário denominada *Escala Funcional de Incapacidade do Pescoço de Copenhagen – EFIPC* ^(1)^.

As alterações vocais impactam na qualidade de vida dos indivíduos. Quando a voz é utilizada como meio profissional, o impacto é ainda mais significante e não depende do grau da disfonia^(3)^. Assim sendo, mesmo que mínimo, a pessoa com problema de voz terá como consequência piora da sua qualidade de vida e desempenho profissional^(4)^.

Algumas disfunções da região cervical podem acarretar dores e afetar as funções desempenhadas por vários grupos musculares e sistemas, dentre eles os músculos anteriores do pescoço e a laringe e, consequentemente, podem resultar em modificações no trato vocal e na produção da voz^(5,6)^. Concomitantemente, estudos demonstram que alguns tipos de disfonias também estão relacionadas às modificações musculares que podem provocar desconforto e dor cervical^(6)^.

A musculatura do pescoço está diretamente relacionada à musculatura extrínseca e intrínseca da laringe, ocasionando alterações vocais e alterações da função cervical em situações de esforço fonatório excessivo e repetitivo^(7)^.

A tensão e a dor muscular da região cervical afetam diretamente a musculatura extrínseca da laringe, e consequentemente altera os músculos intrínsecos da mesma, logo a funcionalidade e a estrutura das pregas vocais são alteradas, fazendo com que o indivíduo sofra com alguma disfonia^(8,9)^.

Além disso, a literatura^(10)^ apresenta evidências de que a tensão na região da cintura escapular e a cervicalgia são queixas comuns em indivíduos disfônicos, demonstrando que essas alterações musculoesqueléticas são mais presentes em indivíduos com disfonias do que em indivíduos sem queixas vocais.

A disfonia por tensão muscular (DTM)^(7)^ é caracterizada como uma alteração na qualidade da voz decorrente de um excessivo esforço fonatório. Este esforço fonatório se caracteriza como uma hiperfunção muscular compensatória que altera a funcionalidade e o desempenho laríngeo, articulatório, ressonantal, respiratório e cervical, levando a uma DTM^(7)^.

Muitos fatores podem desencadear a DTM, principalmente o comportamento vocal abusivo, tornando-a um distúrbio compensatório para uma disfunção laríngea^(11)^.

A literatura pesquisada^(12)^ demonstra estar atenta em buscar os fatores associados aos sintomas vocais e laríngeos em indivíduos disfônicos. Porém, são poucos estudos que relacionam disfonias com cervicalgia, apesar de ser um sinal clínico encontrado com frequência em indivíduos com alterações vocais^(12,13)^.

Pesquisas consideram os profissionais da voz disfônicos como um grupo com mais queixas relacionadas à dor e ao desconforto cervical do que profissionais da voz sem queixas vocais^(13)^. Os profissionais da voz geralmente estão mais suscetíveis a desenvolverem sinais e sintomas vocais e cervicais negativos devido à maior demanda vocal, maior exigência respiratória, e postura corporal específica, fatores estes associados ao tipo da atuação profissional^(14)^.

Ademais, estudos levantam a hipótese de que, quando comparados a indivíduos sem queixas vocais, indivíduos disfônicos apresentam maior intensidade e constância de desconforto na região cervical e cervicalgia, apresentando um maior impacto negativo na qualidade de vida^(10)^.

Desta forma, esta pesquisa visa analisar se mulheres cantoras disfônicas apresentam maior ocorrência de queixas de desconforto e dor cervical do que cantoras sem queixas vocais autorreferidas.

## MÉTODO

Trata-se de um estudo observacional analítico transversal, aprovado pelo Comitê de Ética em Pesquisa da Universidade Federal de Minas Gerais (UFMG) sob o número 59014916.6.0000.5149.

Para desenvolver a pesquisa, foram selecionadas 62 cantoras divididas em dois grupos:

1. Grupo 1 (G1): cantoras disfônicas com presença de alteração vocal definida por avaliação fonoaudiológica, e presença de alteração laríngea definida por avaliação otorrinolaringológica (n=30);
2. Grupo 2 (G2): cantoras sem queixas vocais autorreferidas (n=32).

Foram critérios de inclusão para a pesquisa ser cantora popular, amadora ou profissional, entre 18 e 55 anos. Para o grupo de cantoras disfônicas (G1) os critérios de inclusão foram apresentar alteração na qualidade da voz, queixa vocal, e alteração laríngea. Para o grupo de cantoras sem queixas vocais autorreferidas (G2) foram critérios de inclusão não apresentar queixa de alteração vocal e de sintomas vocais.

Foram critérios de exclusão estar em período menstrual ou pré-menstrual; apresentar infecções de vias aéreas superiores; e estar grávida no momento da coleta dos dados.

Para avaliação da qualidade vocal do grupo G1 foi realizada análise perceptivo-auditiva por consenso por dois fonoaudiólogos com mais de 10 anos de experiência na clínica vocal. Foi analisado o parâmetro perceptivo-auditivo de grau geral da disfonia em uma escala de quatro pontos, sendo zero para qualidade vocal neutra e 3 para grau de alteração intenso.

Para a avaliação otorrinolaringológica do G1, as participantes foram submetidas ao exame de videolaringoscopia de alta velocidade (High-speed Videoendoscopy- HVS) realizado com o equipamento: CHSV (*Color High-Speed Video)*, modelo 9710 da marca Kay Pentax® (Kay Elemetrics Corporation, Lincoln Park, NJ, USA). Os exames foram realizados e analisados por dois médicos otorrinolaringologistas por consenso. A alteração laríngea foi definida a partir da presença de alteração da coaptação glótica e/ou de lesões nas pregas vocais.

Para analisar a presença/ausência das queixas vocais autorreferidas pelas cantoras de ambos os grupos (G1 e G2), foram aplicadas três questões sobre a autopercepção vocal:

1. Você tem alguma dificuldade ou desconforto na sua voz cantada?
2. Você tem alguma dificuldade ou desconforto na sua voz falada?
3. Você acha que sua voz é alterada?

A presença de queixa vocal foi definida pelas respostas afirmativas às três perguntas, e a ausência de queixa vocal pela negativa nas três perguntas.

O G1 foi composto por 30 cantoras disfônicas com idade de 21 a 54 anos (média= 30,33, DP= 7,17), sendo 18 (60,0%) cantoras amadoras e 12 (40,0%) cantoras profissionais, com qualidade vocal alterada, sendo 26 com grau de alteração leve, e quatro com grau moderado. Na avaliação laríngea, 19 (63,33%) cantoras apresentaram alterações com os seguintes diagnósticos: fenda triangular médio-posterior (N=9); nódulos de pregas vocais (N=7); cisto de prega vocal (N=2) e fenda fusiforme anterior (N=1).

O grupo de cantoras sem queixas vocais (G2) foi composto por 32 cantoras com idade de 19 a 55 anos (média= 29,1, DP= 9,89), sendo 17 (53,1%) cantoras amadoras e 15 (46,9%) cantoras profissionais.

Para este estudo, considerou-se cantora profissional aquela que exercia atividade remunerada para esta função^(15)^.Os grupos foram pareados por idade, sem diferença entre eles (p=0,13).

### Escala Funcional de Incapacidade do Pescoço de Copenhagen – EFIPC

O questionário EFIPC, é um protocolo de autoavaliação, adaptado para o português brasileiro^(16)^ composto por 15 perguntas, sendo cinco de direção positiva e 10 de direção negativa, relacionadas à dor cervical e foi utilizado nesse estudo para avaliar e mensurar em qual intensidade ela é prejudicial à qualidade de vida, funcionalidade e desempenho diário das cantoras.

As questões de 1 a 5 do questionário, são de direção positiva, o que significa que se a cantora responder “sim”, indica que ela apresenta uma adequada condição cervical. Nestas questões, as respostas são pontuadas da seguinte forma: a opção “sim” corresponde a 0 ponto; “às vezes” corresponde a 1 ponto, “não” corresponde a 2 pontos e “não se aplica” não há valor correspondente (-). Já as questões de 6 a 15, são questões de direção negativa, o que significa que se a cantora responder “sim”, indica que ela apresenta uma condição cervical inadequada. As respostas, com suas respectivas pontuações são: a opção “sim” corresponde a 2 pontos, “às vezes” corresponde a 1 ponto, “não” corresponde a 0 pontos e “não se aplica” não há valor correspondente (-). Diante disso, a pontuação máxima que pode ser obtida no EFIPC é de 30 pontos, indicando que quanto maior for a pontuação, maior será a disfunção cervical^(16)^.

As cantoras disfônicas (G1) assinaram o Termo de consentimento livre e esclarecido (TCLE), e responderam às questões sobre queixas vocais e o EFIC de forma presencial. As cantoras sem queixas vocais (G2) assinaram o TCLE e responderam os dois questionários de forma virtual, por meio da ferramenta *Google Forms*. Estas diferenças na aplicação dos questionários entre os dois grupos foram decorrentes do início da pandemia causada pelo coronavírus (SARS-COV-2).

### Análise estatística

A análise estatística dos dados foi realizada por meio do programa estatístico MINITAB versão 17. Primeiramente foi realizada uma análise descritiva dos dados com medidas de tendência central e dispersão. Posteriormente, foi utilizado o teste de Anderson-Darling para verificar a normalidade da amostra. Para comparação entre os grupos utilizou-se o teste não paramétrico de Mann-Whitney. Considerou-se o nível de confiança de 95%.

## RESULTADOS

A Tabela 1 apresenta os dados do questionário EFIPC de ambos os grupos analisados: cantoras disfônicas e cantoras sem queixas vocais autorreferidas. Os resultados sinalizam que as cantoras disfônicas, quando comparadas às cantoras sem queixas vocais, obtiveram maior pontuação final no questionário, indicando que elas apresentam mais queixas e limitações funcionais e diárias devido a dor cervical.

**Tabela 1:** Comparação do escore total do questionário EFIPC entre ambos os grupos

	**Mínimo**	**Máximo**	**Média**	**Mediana**	**DP**	**p-valor**
**G1**	0,0	24,0	7,4	6,0	5,8	
**G2**	0,0	14,0	2,7	1,0	4,05	0,0001

**Legenda:** G1 – cantoras disfônicas; G2 – cantoras sem queixas vocais autorreferidas; DP – desvio padrão

## DISCUSSÃO

A presença de disfonia pode estar associada a desconfortos laríngeos que alteram a fonação, além de desconforto emocional e social, que interferem nas atividades de vida diária dos indivíduos^(15-17)^.

Considerada de natureza multifatorial^(11)^, a DTM pode estar associada e uma hiperfunção muscular para compensar a insuficiência glótica do indivíduo, exigindo maior esforço e compensação vocal durante a fonação. Estudos anteriores^(13,14)^ revelam que profissionais da voz podem apresentar um maior número de sinais e sintomas vocais negativos devido à intensa demanda de voz, que pode acarretar fadiga vocal e, consequentemente, maior tensão e dor muscular na região cervical devido a compensações funcionais negativas.

Por apresentarem uma anatomia laríngea de menor tamanho, associada à uma voz mais aguda, as mulheres apresentam maior desenvolvimento de alterações vocais e problemas cervicais^(12)^.

Os resultados da presente pesquisa indicam que cantoras disfônicas apresentam mais queixas e limitações funcionais e diárias devido a dor cervical quando comparadas a cantoras sem queixas vocais. Tais resultados são apoiados pela literatura que evidencia que as mulheres disfônicas referem mais dores laríngeas, cervicais, e dorsais do que as mulheres sem queixas vocais^(6)^. Além disso, pesquisas^(6,12)^ sugerem que mulheres disfônicas tendem a apresentar encurtamento da musculatura cervical devido à hiperfunção muscular, e presença de disfonia decorrente de mau uso e abuso vocal^(6,12)^.

Como mostra a literatura^(5)^, alterações na produção vocal podem acarretar em disfunção da musculatura anterior do pescoço, como os músculos supra e infra-hióideos, escalenos e esternocleidomastóideos. Indivíduos disfônicos podem apresentar dor cervical tanto no repouso quanto durante a fonação, além de uma maior hiperatividade na musculatura envolvida nessa função^(18)^, acarretando em alterações de postura e diminuição dos movimentos da cintura escapular^(19,20)^. Alterações de postura envolvendo a região cervical podem alterar a posição da cabeça e do pescoço, levando a alterações funcionais nas estruturas presentes nessas regiões, favorecendo o aparecimento de modificações no trato vocal e, consequentemente, na qualidade da voz dos indivíduos^(21,22)^.

A voz é fundamental na vida dos indivíduos que a usam como instrumento de trabalho^(23)^ principalmente no caso dos profissionais que fazem uso da voz de forma artística, pois estes apresentam uma maior demanda vocal e ajustes mais refinados em sua produção^(3)^. Avaliar o grau de incapacidade cervical em cantoras disfônicas é importante para um melhor direcionamento do processo terapêutico na clínica vocal.

Como limitações da pesquisa podemos apontar o fato de o grupo de cantoras sem queixas vocais ter sido caracterizado apenas pela presença/ausência de queixas vocais na voz falada e/ou cantada. Esta limitação deu-se devido às restrições apresentadas pela pandemia causada pelo coronavírus, o que impossibilitou a avaliação presencial das mesmas. Vale ressaltar que a literatura indica que cantores apresentam uma refinada autopercepção vocal, além de uma maior consciência e preocupação com suas vozes e, por isso, um distúrbio de voz, por menor que seja, torna-se significativo a ponto de afetar sua qualidade de vida e sua ocupação, caracterizando a presença de queixa vocal autorreferida^(24)^. Ademais, os cantores, por usarem a voz no dia a dia como instrumento profissional, conseguem relatar com mais minúcia as alterações vocais e corporais percebidas^(3,13,14)^.

Outra limitação está relacionada ao fato das participantes não serem avaliadas quanto à habilidade de tocar instrumentos musicais, o que também pode impactar na postura corporal e desencadear alterações e queixas cervicais uma vez que, segundo a literatura, 84% dos músicos relatam apresentar algum tipo de queixa musculoesquelética^(25)^. Ressalta-se também que este estudo avaliou e evidenciou a associação entre a disfonia e queixas cervicais em cantoras disfônicas e não disfônicas, porém, novos estudos que verifiquem a relação de causalidade são necessários.

Considerando que os protocolos de autoavaliação não se constituem classificadores perfeitos e não devem ser utilizados de forma isolada, o EFIPC mostrou-se um instrumento útil e de boa aplicabilidade na clínica fonoaudiológica, podendo contribuir na avaliação da funcionalidade cervical em cantoras disfônicas.

## CONCLUSÃO

O presente estudo demonstrou que cantoras disfônicas apresentam mais dor e desconforto cervical do que cantoras sem queixas vocais autorreferidas, caracterizando uma maior incapacidade cervical.
